# A high-throughput pipeline for designing microarray-based pathogen diagnostic assays

**DOI:** 10.1186/1471-2105-9-185

**Published:** 2008-04-10

**Authors:** Ravi Vijaya Satya, Nela Zavaljevski, Kamal Kumar, Jaques Reifman

**Affiliations:** 1 Biotechnology HPC Software Applications Institute, Telemedicine and Advanced Technology Research Center, US Army Medical Research and Materiel Command, Fort Detrick, MD 21702, USA

## Abstract

**Background:**

We present a methodology for high-throughput design of oligonucleotide fingerprints for microarray-based pathogen diagnostic assays. The oligonucleotide fingerprints, or DNA microarray probes, are designed for identifying target organisms in environmental or clinical samples. The design process is implemented in a high-performance computing software pipeline that incorporates major algorithmic improvements over a previous version to both reduce computation time and improve specificity assessment.

**Results:**

The algorithmic improvements result in significant reduction in runtimes, with the updated pipeline being nearly up to five-times faster than the previous version. The improvements in specificity assessment, based on multiple specificity criteria, result in robust and consistent evaluation of cross-hybridization with nontarget sequences. In addition, the multiple criteria provide finer control on the number of resulting fingerprints, which helps in obtaining a larger number of fingerprints with high specificity. Simulation tests for *Francisella tularensis *and *Yersinia pestis*, using a well-established hybridization model to estimate cross-hybridization with nontarget sequences, show that the improved specificity criteria yield a larger number of fingerprints as compared to using a single specificity criterion.

**Conclusion:**

The faster runtimes, achieved as the result of algorithmic improvements, are critical for extending the pipeline to process multiple target genomes. The larger numbers of identified fingerprints, obtained by considering broader specificity criteria, are essential for designing probes for hard-to-distinguish target sequences.

## Background

Recent developments in technology have led to the sequencing of many eukaryotic and prokaryotic organisms. Availability of these genomic sequences unlocked opportunities for the development of whole-genome-based diagnostic assays, such as DNA microarrays and polymerase chain reaction (PCR) assays, which offer higher specificity than traditional methods based on a single gene or protein [1]. Because of their simplicity and efficiency, these assays are increasingly being used for various applications in medicine, environmental monitoring, and biodefense. The popularity of these assays, in turn, triggered the development of different computational tools for sequence-based signature design [1-4].

Microarray-based pathogen diagnostic assays are gaining popularity due to their ability to test for hundreds, or even thousands, of pathogens in a single diagnostic test [5]. Wilson *et al*. [6] used 50-thousand 20 mer overlapping oligonucleotides to detect 18 pathogens, Wang *et al*. [7] reported using a microarray with 11-thousand 70 mer oligonucleotides that can identify ≥ 954 distinct viruses, while Palacios *et al*. [8] designed a panmicrobial microarray comprising nearly 30-thousand 60 mer probes. Current technology for custom microarray design enables up to 385000 oligonucleotides per slide, while arrays with 2.1 million probes are available for other high-throughput applications, such as comparative genome hybridization [9]. The ability to simultaneously screen against a wide range of targets is essential for detecting biological threat agents in environmental samples, where there may be no prior knowledge about the specific pathogens likely to be present in the sample.

In terms of the computational problems, the design of microarray probes for pathogen identification is fundamentally different from the design of microarray probes for gene expression analysis. An oligonucleotide probe designed for monitoring the expression of a gene should hybridize only to the mRNA of the corresponding gene, and should not have any significant cross-hybridization with other mRNAs from the same organism. Because all sequences involved are gene transcripts of a single organism, the combined length of the sequences is typically, at most, a few megabases. The problem, though computationally intensive, can be handled by a single processor in less than an hour [10]. Many efficient computational tools have been developed for designing microarray probes for gene expression analysis [10-15].

In contrast, oligonucleotide probes designed for pathogen diagnostic assays should hybridize only with the intended target and should not have any significant cross-hybridization with any nontarget genome. This necessitates the comparison of each candidate probe against every available sequenced nontarget genome. To date, the combined size of the nucleotide sequences in the National Center for Biotechnology Information (NCBI) *nt *database is greater than 21 gigabases and is bound to grow exponentially as more and more genomes are sequenced. The amount of sequence data that need to be analyzed for each target pathogen is also increasing due to multiple strains of many pathogens being sequenced. Therefore, the sheer magnitude of the search space necessitates efficient high-throughput algorithms that both quickly scan large genomic databases and reduce the space to a small set of unique regions in the target genome.

In this paper, we present a software tool for identifying oligonucleotide fingerprints for microarray-based pathogen diagnostic assays. The software, named Tool for Oligonucleotide Fingerprint Identification (TOFI), is an integrated, scalable, high-performance-computing pipeline that combines genome comparison tools, probe design software, and sequence alignment programs in order to design highly specific microarray probes for a given target pathogen (i.e., one complete genomic sequence). This pipeline (henceforth referred to as TOFI-beta), is an improvement over an earlier version of the pipeline (TOFI-alpha), presented by Tembe *et al*. [4]. TOFI-beta incorporates several optimizations and enhancements that significantly reduce the overall execution time of the pipeline, opening up possibilities for future extension of the system to design fingerprints common to a group of targets. In addition, TOFI-beta uses new, multiple criteria for estimating probe specificity, which, for any given genome, results in a considerable increase in the number of identified *in silico *fingerprints without increase in the (expected) false-positive rate. This is particularly important in cases where the target sequence is a close match to other sequences, where the larger number of *in silico *fingerprints increases the chance of identification of true fingerprints.

### Existing methods for design of pathogen diagnostic assays

One method to design probes is to select regions of the pathogen genome that are known to be associated with specific functions. For example, specific genes of bacterial genomes, such as the 16S rRNA gene [16], virulence genes [17], and antibiotic resistance genes [18], have all been used to design microarray probes for species-level diagnostics.

Another method to design probes is to employ the whole genome of the pathogen. A few software tools/algorithms have been proposed to guide the design of whole-genome-based pathogen diagnostic assays [1-4,11-14]. Some of these tools are intended for microarray-based assays [2,4,11-14], whereas others are intended for PCR-based assays [1,3]. Most of these tools, however, do not have the capability of testing for specificity against a large number of nontarget genomes; they are based on the assumption that the signatures need only be unique with respect to the host or a small set of nontarget sequences. For instance, Kaderali and Schliep [2] developed an algorithm that analyzes a set of input target sequences and designs a single probe for each target, with the probe being unique with respect to all other input sequences. The uniqueness of the probe is determined by constructing a generalized suffix tree for all the input target sequences. The method presented by Putonti *et al*. [19] designs probes that are unique with respect to a host organism. Both of these approaches are clearly not adequate if the signatures are to identify pathogens from environmental samples containing a multitude of nontarget organisms. Because, in general, there is no prior knowledge of the contaminants in a sample, the signatures have to be unique with respect to all known nontarget sequences.

The KPATH pipeline for PCR assays [1] is the seminal software that introduced the concept of *in silico *comparison against all known nontarget sequences. Insignia [3] is another tool for designing PCR assays. Unlike the current version of TOFI, which designs fingerprints for a single genome, both KPATH and Insignia have the ability to design fingerprints that are common to multiple target genomes. To our knowledge, KPATH and Insignia are the only tools other than TOFI [4] that have the provision for *in silico *sequence-based testing for specificity against multiple nontarget genomes.

Neither KPATH nor Insignia is applicable for designing microarray fingerprints, as the design and specificity requirements of microarray fingerprints are quite different from those of PCR signatures. For example, the most commonly used PCR signatures consist of a probe and two primers, which, due to their short length [18–25 base pairs (bp)] and constraints on the inter-primer distance, can tolerate inexact matches with nontarget sequences without much degradation in specificity. Conversely, in addition to being characterized by only one DNA segment with no spacing constraints, microarray probes are generally longer and more susceptible to cross-hybridization even in the absence of an exact match [20]. This requires more extensive searches, for both exact and inexact matches, against nontarget sequences to identify highly specific fingerprints for microarrays.

## Implementation

**Figure 1:**
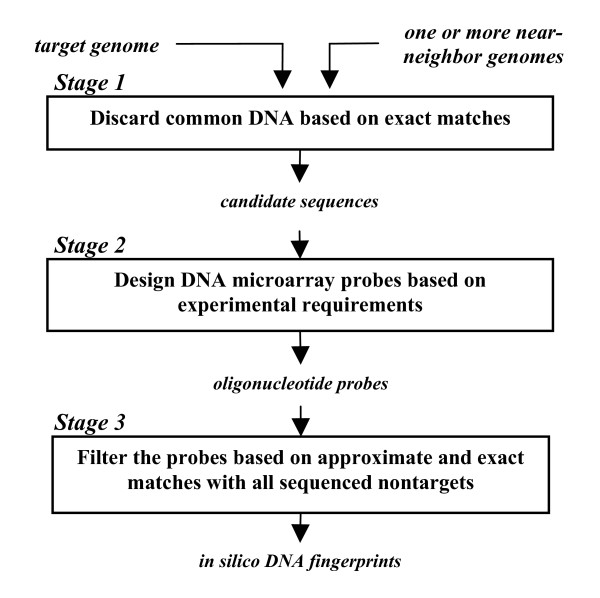
**The three stages of the TOFI-beta pipeline**. On average, about 90% of the total computation time is spent in Stage 3.

The TOFI pipeline consists of the three main stages illustrated in Figure 1. The stages are designed so that large portions of the target genome are eliminated in the less-expensive two initial stages, and the computationally more expensive searches for specific fingerprints are performed over smaller regions of the target genome in the final stage.

The first stage uses the *suffix-tree*-based MUMmer [21] program to perform pairwise comparisons of the target genome with each nontarget genome and eliminates regions in the target genome that have exact matches with one of the nontarget genomes. The surviving regions of the target genome, called *candidate sequences*, are then passed on to the second stage of the pipeline. Given a pair of sequences, MUMmer finds all maximal matches that are at least as long as a threshold (termed *minmatch*) between the two sequences. TOFI uses MUMmer to find these maximal matches, and eliminates regions in the target genome that are covered by these maximal matches. The threshold to use for *minmatch *is calculated based on the specificity parameters supplied by the user. This ensures that every segment of the target genome that is at least as long as the minimum probe length and satisfies the specificity parameters is part of a candidate sequence.

Stage 2 identifies oligonucleotides of desired length from the candidate sequences that satisfy experimental conditions, such as melting temperature (*T*_*m*_) and GC content. TOFI uses the Oligonucleotide Modeling Platform (OMP) software [22] to identify these oligonucleotides, also referred to here as probes. OMP uses the nearest neighbor hybridization model [23] to calculate *T*_*m *_and to estimate if a probe forms any secondary structures that may prevent it from hybridizing to the target genome.

In the third and final stage of the pipeline, TOFI performs a BLAST [24] search against a local copy of the NCBI *nt *database for each probe. The program uses mpiBLAST [25] to run BLAST in parallel on multiple processors. Probes with alignments to nontarget genomes that exceed the specificity thresholds are eliminated, and the surviving probes become the *in silico *DNA fingerprints for the target genome. These probes are then subjected to experimental validation to test their sensitivity and specificity.

TOFI-beta incorporates major modifications in the first and third stages of the pipeline to increase computational speed and enhance the specificity assessment of the fingerprints. In the following, we compare TOFI-beta and TOFI-alpha and describe these improvements.

### Improvements in Stage 1

In Stage 1, the major improvement in TOFI-beta over TOFI-alpha is the comparison of the target against *multiple *nontarget genomes for finding exact matches. TOFI-alpha only allows for comparison against a single nontarget sequence. Comparison against a single genome is effective in eliminating a large portion of the target genome when a closely-related, nontarget near-neighbor genome sequence is available. However, when such a nontarget sequence is not available, too many candidate sequences are passed on to the later stages of the pipeline, which are computationally more expensive. Even when a closely-related, nontarget near-neighbor is available, comparisons against additional nontarget genomes is advantageous. As Stage 1 is relatively inexpensive, the additional time spent in this stage is offset by the much larger reduction in computation time in the later steps, yielding a very favorable trade-off in overall runtime. Potentially, the target genome could be compared with all nontarget sequences in the entire *nt *database.

As described by Tembe *et al*. [4], sequence comparisons for exact matches in Stage 1 are performed using the *nucmer *module in MUMmer. Some modifications were required in TOFI-beta, however, to avoid some performance issues when using a large database of nontarget sequences. Since the larger databases are too big for MUMmer, they are split into smaller databases and the target sequence is compared for exact matches against the smaller databases. This procedure is parallelized in TOFI-beta so that the target sequence is compared against a different set of nontarget sequences at each processor. The results are then assembled and processed so that only unique regions of the target genome are passed on to the next stage.

### Improvements in Stage 3

In Stage 3, each probe that is generated in Stage 2 is screened for cross-hybridization against all available nontarget genome sequences in the *nt *database using BLAST [24]. Stage 3 is, by far, the most computationally expensive stage, which takes about 99% of the total runtime of TOFI-alpha.

**Figure 2:**
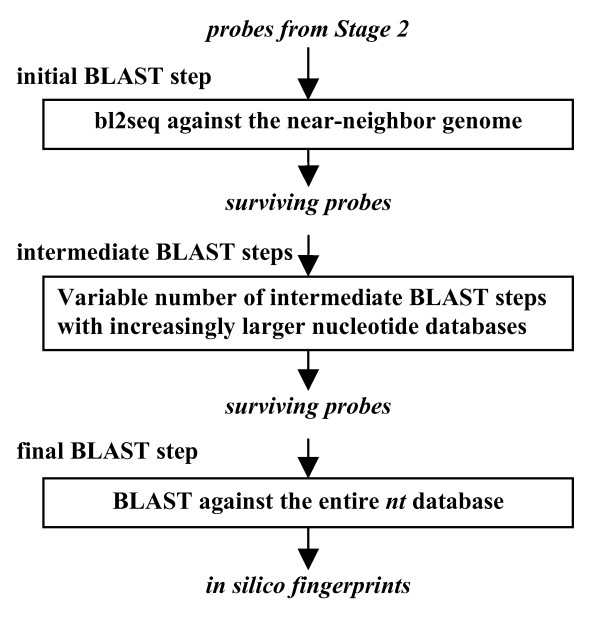
**Stage 3 of TOFI-beta**. The initial BLAST step is optional. It is bypassed if a near-neighbor genome is not available. The databases used in the intermediate BLAST steps must consist of organisms that are closely related to the target pathogen.

In TOFI-alpha, Stage 3 consists of a single step in which a BLAST search is performed for each probe against the complete *nt *database. In contrast, as illustrated in Figure 2, the third stage in TOFI-beta consists of multiple, hierarchical BLAST steps, with the computational cost of the BLAST searches increasing with the number of steps. At each step, the oligonucleotide probes having significant alignments with nontarget sequences are removed, and only the surviving probes are passed on to the next, more expensive step. In the first step, we use the pairwise BLAST program bl2seq to identify matches with a near-neighbor genome, and the probes that meet the specificity requirements are passed on to the subsequent steps. In the absence of a near-neighbor genome, this step is bypassed.

The subsequent steps consist of a series of BLAST searches using *blastn*, where at each step the probes are queried against increasingly larger nucleotide databases of more distantly-related organisms to the target organism. For example, when *Yersinia pestis *is the target organism, the probes are first queried against databases consisting of sequences of Proteobacteria, then all other bacteria, and finally the *nt *database. Because the time taken to perform a BLAST search increases with database size and a probe is more likely to match sequences of closely-related organisms, the strategy in TOFI-beta is to perform relatively less expensive BLAST searches against small databases of related organisms first, eliminating many nonspecific probes before performing more comprehensive and costly BLAST searches. The hierarchical sequence databases are manually constructed. The probes that meet the specificity criteria in all BLAST steps are provided as the *in silico *DNA fingerprints for the target organism.

### Improved specificity criteria

Probes designed for pathogen identification have to be unique to the target organism, and should not cross-hybridize with any nontarget organism. High sequence similarity between a probe and a nontarget sequence, apparent from the presence of good pairwise sequence alignments, is generally indicative of cross-hybridization between the two. There are multiple criteria for determining the specificity of a probe: overall sequence similarity, contiguous matches, and predicted free energy have all been shown to be important measures of the potential for cross-hybridization [26]. In addition to these criteria, we propose the use of near-contiguous matches (i.e., long stretch of matches with very few mismatches, insertions or deletions) to measure probe specificity.

Sequence similarity, i.e., the number of matches (or mismatches) in the alignment between two sequences, is one criterion for estimating cross-hybridization. This criterion measures only the matching bases. That is, it does not take into consideration how the matches are distributed in the alignment. TOFI-alpha uses the number of mismatches in the alignment, denoted by *T*, as the sole criterion for determining probe specificity. However, when the probe length is variable, specifying the matching bases as a percentage of the probe length allows for a more consistent measure of similarity, as a single threshold can be used for various probe lengths. The percentage of matching bases in the alignment between two sequences is commonly referred to as the identity of the two sequences. In TOFI-beta, we use sequence identity as one of several criteria for estimating cross-hybridization.

In order to incorporate contiguous and near-contiguous matches in determining probe specificity, we use a series of thresholds *M*_0_, *M*_1_, *M*_2_, and *M*_3_, where *M*_*i *_is the maximum length of a contiguous region in which the alignment between a probe and a nontarget sequence has (*M*_*i *_- *i*) matches and *i *mismatches/insertions/deletions. Accordingly, *M*_0 _is the length of the longest stretch of contiguous matches between a probe and a nontarget genome.

The use of multiple criteria for specificity is deemed to yield a number of advantages. First, other factors, apart from overall sequence identity, influence the hybridization of a probe to a nontarget sequence. Kane *et al*. [27] performed empirical analysis on 50 mer oligonucleotides in order to measure, among other things, the effect of overall sequence identity and contiguous stretches of similarity on cross-hybridization. They concluded that a probe is likely to cross-hybridize with a nontarget if overall sequence identity is > 75% or if there is a contiguous match > 15 bp. Li *et al*. [26] also concluded that better specificity can be obtained by using multiple criteria, such as sequence identity, length of contiguous matches and hybridization energy.

Second, the use of multiple criteria for specificity gives finer control; one can relax the threshold value for each individual criterion and yet obtain more *in silico *fingerprints with comparable specificity to fingerprints that can be obtained using a single criterion. Conversely, using a single criterion does not give much control over the specificity of the selected probes. Applying strict thresholds for a single criterion might result in missing many specific probes, whereas relaxing the thresholds may lead to too many nonspecific probes [20].

An additional advantage is that using multiple specificity criteria, including contiguous matches, improves runtime. The selection of specificity criteria affects the choice of the length of minimum exact matches in Stage 1, and using multiple criteria allows for the selection of a smaller threshold for minimum exact matches. This selection results in fewer candidate sequences passing Stage 1, thereby improving the overall pipeline performance.

**Figure 3:**
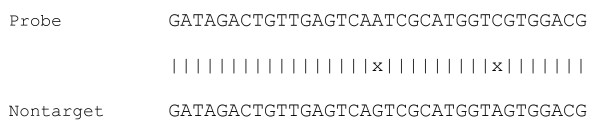
**An alignment with a long near-contiguous match**. In the alignment, the longest contiguous match is only 17 bp, but the longest near-contiguous match with one mismatch is 27 bp.

The thresholds *M*_1_, *M*_2_, and *M*_3 _help design robust fingerprints that are not affected by small variations in nontarget sequences. Using *M*_1_, *M*_2_, and *M*_3 _one can avoid situations in which a small number of mutations/insertions/deletions in a nontarget sequence might potentially lead to long stretches of contiguous matches between the probe and nontarget, causing the probe to cross-hybridize with the nontarget. For instance, in the example shown in Figure 3, the probe does not have a very long stretch of contiguous matches with a nontarget, but rather a long *near-contiguous *match. In this instance, the probe might cross-hybridize with the nontarget because of the long stretch of near-contiguous match. The threshold *M*_1 _is particularly useful in avoiding regions around common single nucleotide polymorphisms between the target and nontarget genomes.

### Using free energy (ΔG) for probe selection

Li *et al*. [26] have suggested that free energy (ΔG) is an important measure of probe specificity. However, computing the ΔG between a probe and nontarget sequences involves traversing through each nontarget genome and computing ΔG for each alignment with the probe. Given the large number of nontarget genomes and the relatively high computational cost of each ΔG calculation, such an approach is not practical for the current application. A more feasible strategy would be to obtain the BLAST hits for the probe and compute ΔG against each significant hit. However, even this strategy would be impractical, owing to the large number of fingerprints reported at the end of Stage 2. A feasible strategy is to perform the calculations only at the very final stage, after the probes have been screened using other specificity criteria. Hence, ΔG estimation (computed with OMP) is provided as an optional post-processing step in TOFI-beta.

## Results

The TOFI pipeline can design oligonucleotide probes of any length. The results that we present here are for the design of variable-length probes with length varying from 35 to 40 bp. The choice of probe length was solely based on the requirements of the downstream field-deployable microarray platform for which these probes are designed, and hence we do not attempt to find optimal probe lengths for pathogen identification. In general, shorter probes result in better specificity, and longer probes result in better sensitivity [28,29].

### Performance improvements

To compare runtimes of TOFI-beta and TOFI-alpha under similar conditions, we conducted tests with *T *as the only specificity criterion in both, although TOFI-beta can make use of multiple specificity criteria as described above. Table 1 shows comparative runtimes for four different genomes with *T *= 9, which is a practical threshold for probes with lengths between 35 and 40 bp. It can be seen that TOFI-beta is at least twice as fast as and almost as much as five times faster than TOFI-alpha in all test cases. The results in Table 1 are representative of runs with other bacterial pathogens.

**Table 1:** Performance comparison of TOFI-alpha and TOFI-beta

Genome (length in base pairs)	Near-neighbor (length in base pairs)	TOFI-alpha runtime (hrs)	TOFI-beta runtime (hrs)	Improvement
*Yersinia pestis *(4653728)	*Yersinia pseudotuberculosis *(4744671)	11	4	275%
*Francisella tularensis *(1892819)	*Francisella philomiragia *(2049711)	22	5	440%
*Burkholderia mallei *(5835527)	*Burkholderia thailandensis *(6723972)	21	7	300%
*Brucella melitensis *(3294931)	*Agrobacterium tumefaciens *(2074782)	55	21	261%

The runtimes are rounded off to the nearest hour. All runs were carried out on 74 processors.

The performance gains obtained in TOFI-beta are partly due to using more near-neighbor genomes in Stage 1, and partly due to using multiple hierarchical steps in the BLAST stage. Table 2 shows a stage-by-stage comparison for *F. tularensis *using 74 processors. In this case, TOFI-beta is nearly five times faster than TOFI-alpha, where about half of the speedup is the result of using multiple nontarget genomes in Stage 1. The changes in Stage 1 reduce the number of probes reaching Stage 3 by 44%, from 33299 in TOFI-alpha to 18668 in TOFI-beta, where the additional time for carrying out these steps (31 minutes) is offset by the much larger reduction in computation time in Stage 3 (nearly 11 hours, not shown in Table 2). The rest of the gains in performance are the result of hierarchical filtering in the BLAST stage. The initial and intermediate inexpensive BLAST steps eliminated 15288 probes out of the 18668 probes in less than two hours, which would have taken more than 10 hours if a single BLAST step were to be used as in TOFI-alpha.

**Table 2:** Comparison of TOFI-alpha and TOFI-beta for the *F. tularensis *genome on 74 processors

		TOFI-alpha		TOFI-beta	
Stage	Step	Time	Output	Time	Output

1	MUMmer with near-neighbor	**1 m**	**1730 **candidates, **1889863 **bp	**1 m**	**1730 **candidates, **1889863 **bp
1	MUMmer with Proteobacteria	-	-	**8 m**	**1882 **candidates, **1768235 **bp
1	MUMmer with *nt *database	-	-	**23 m**	**2140 **candidates **1192259 **bp
2	OMP	**2 m**	**33299 **probes	**2 m**	**18668 **probes
3	bl2seq with near-neighbor	-	-	**5 m**	**6979 **probes
3	BLAST with Proteobacteria	-	-	**1 hr 37 m**	**3380 **probes
3	BLAST with *nt *database	**22 hrs**	**1469 **fingerprints	**2 hrs 13 m**	**1469 **fingerprints
	Total execution time	**22 hrs**		**4 hrs 29 m**	

For both TOFI-alpha and TOFI-beta, *T *= 9 is used as the sole specificity criterion.

The optimum number of BLAST steps depends on many factors, including the size of the target genome, the availability of genomic sequences from closely-related, nontarget genomes, and the number of processors available. Based on empirical evidence obtained by running the pipeline for various bacterial genomes, we observe that the use of one intermediate BLAST step is optimal in reducing the overall runtime. Additional BLAST steps do not reduce the runtime significantly when a large number of processors (> 30) are available. However, when a limited number of processors are available, using additional BLAST steps might be useful in reducing the overall runtime.

### Effect of multiple specificity criteria

Table 3 compares the numbers of *in silico *fingerprints obtained by using *T *versus using sequence identity together with four measures for match contiguity (*M*_0_, *M*_1_, *M*_2_, and *M*_3_). In all four cases, the number of fingerprints obtained is higher using multiple parameters, with the numbers being significantly higher in three out of the four test cases. Empirical analysis on various organisms reveals that a threshold of eight (or 10) for *T *results in too few (or too many) *in silico *fingerprints to test in a single microarray experiment. A threshold of *T *= 9 yields a more reasonable number of fingerprints in most cases. The thresholds for the match contiguity parameters were selected by starting with baseline values recommended in the literature [20,27,30], and making necessary adjustments to obtain a reasonably large number of fingerprints. Thresholds for identity and contiguous matches (*M*_0_) are based on the values suggested by Kane *et al*. [27] for 50 mer oligonucleotides. We find that the thresholds suggested, identity = 75% and *M*_0 _= 15, are too strict, with few probes passing these thresholds when matches with all nontarget genomes are taken into consideration. Therefore, we relaxed the thresholds to identity = 80% and *M*_0 _= 18. The thresholds of 22, 26, and 30 for near-contiguous matches *M*_1_, *M*_2_, and *M*_3_, respectively, were selected based on the threshold used for *M*_0_. We expect that relaxing the thresholds for identity and contiguous matches will not decrease the specificity of the fingerprints reported, because the thresholds for near-contiguous matches (*M*_1_, *M*_2_, and *M*_3_) help eliminate some nonspecific probes that satisfy the thresholds for identity and contiguous matches.

**Table 3:** The number of fingerprints obtained using different specificity criteria for four different target organisms

Genome	*T *= 9	identity = 80%, *M*_0_-*M*_1_-*M*_2_-*M*_3 _= 18-22-26-30
*Yersinia pestis*	614	836
*Francisella tularensis*	1469	2028
*Burkholderia mallei*	572	1146
*Brucella melitensis*	1352	7659

### Case studies: *Francisella tularensis *and *Yersinia pestis*

In the following, we present a detailed comparative analysis of the number and specificity of fingerprints obtained by using individual versus multiple specificity criteria. Our aim is to consider all potential probes that satisfy the experimental constraints and evaluate the specificity criteria based on estimated ΔG of these probes with nontarget genomes. Accordingly, we evaluated all the probes resulting from Stage 2, where in Stage 1 we compared the target with a single near-neighbor genome using an exact match threshold of 32 bp. The results presented here are for *F. tularensis *SCHU S4 (NCBI accession no. NC_006570) and *Y. pestis *CO92 (NCBI accession no. NC_003143). A contig from *F. philomiragia *ATCC 25017 [31] was used as the near-neighbor for *F. tularensis*, and *Y. pseudotuberculosis *IP 32953 (NCBI accession no. NC_006155) was used as the near-neighbor for *Y. pestis*.

Probe design on the candidate sequences, taking into consideration the experimental constraints (probe length between 35 and 40 bp, *T*_*m *_between 70 and 100°C and GC content between 45 and 50%), yielded a total of 33299 probes for *F. tularensis *and 19810 probes for *Y. pestis*. BLAST searches were performed on these probes against the entire *nt *database downloaded from NCBI in July 2007. BLAST hits with the corresponding targets and synthetic constructs were ignored. The remaining BLAST hits were extended on either side by 50 bp, and ΔG with the corresponding probe was estimated using the simulation feature of the OMP software. For each probe, the largest negative ΔG value among all BLAST hits was taken as a conservative measure of cross-hybridization between the probe and a nontarget sequence.

The probes are categorized into *good*, *bad*, or *gray *based on their ΔG values, as actual experimental hybridization results are not available for these probes. Good probes are expected to have little or no cross-hybridization with nontarget genomes, bad probes are expected to have significant cross-hybridization, and the behavior of gray probes is too uncertain to categorize either way. Estimated ΔG should not, however, be construed as a substitute for experimental hybridization tests. We heuristically selected well-spaced ΔG thresholds for good and bad probes in order to assess the relative performance of the different specificity criteria. Probes with ΔG greater than or equal to -16 kcal/mol are categorized as good probes. This threshold was selected because it corresponds to about 50% of the mean ΔG between a probe and its complement, which is the approximate ratio recommended for 50 mers [20]. Probes with ΔG less than -20 kcal/mol (less than 60% of the mean ΔG between a probe and its complement) are considered as bad probes. The probes with ΔG between -20 and -16 kcal/mol are labeled as gray probes. Further increase in separation between good and bad probes is not convenient because it reduces the sample size for comparative assessment.

**Figure 4:**
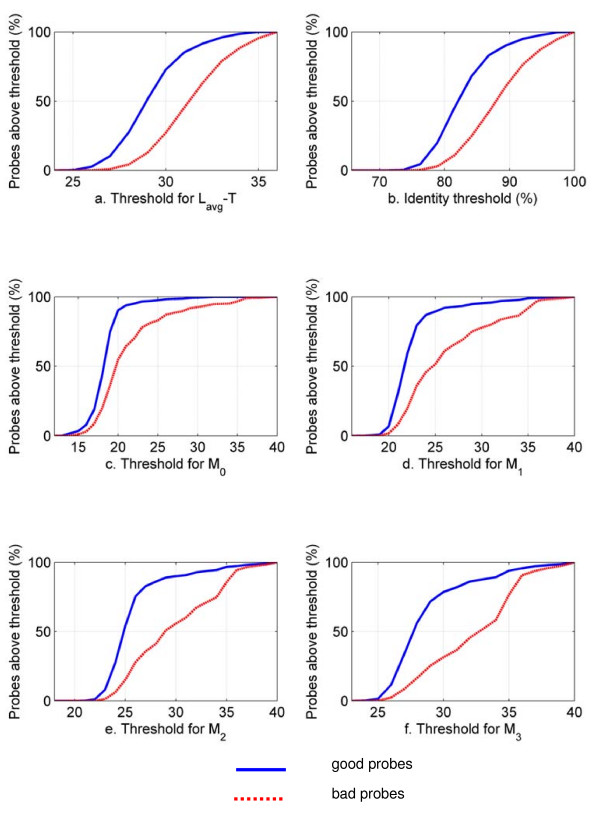
**Percentages of good and bad probes for different individual specificity criteria for *F. tularensis***. Identity and *T *(number of mismatches, presented in Figure 4a as the difference between the average probe length *L*_*avg *_and *T*) are different ways of measuring sequence similarity. *M*_0 _is the maximum length of contiguous matches, and *M*_1_, *M*_2_, and *M*_3 _are measures of near-contiguous matches. It can be seen that none of the thresholds for the individual criteria are effective in clearly discriminating between good and bad probes.

Figure 4 shows the variation of the number of good and bad probes for *F. tularensis *as a function of thresholds for six different individual specificity criteria. It can be seen that, for each individual criterion, the percentage of good probes increases sharply within a narrow range of thresholds. In most cases, the percentage of bad probes closely follows the percentage of good probes, with the effect that none of the thresholds for the individual criteria are helpful in admitting a large percentage of good probes while simultaneously rejecting a large percentage of bad probes. We obtained similar results for *Y. pestis*.

**Figure 5:**
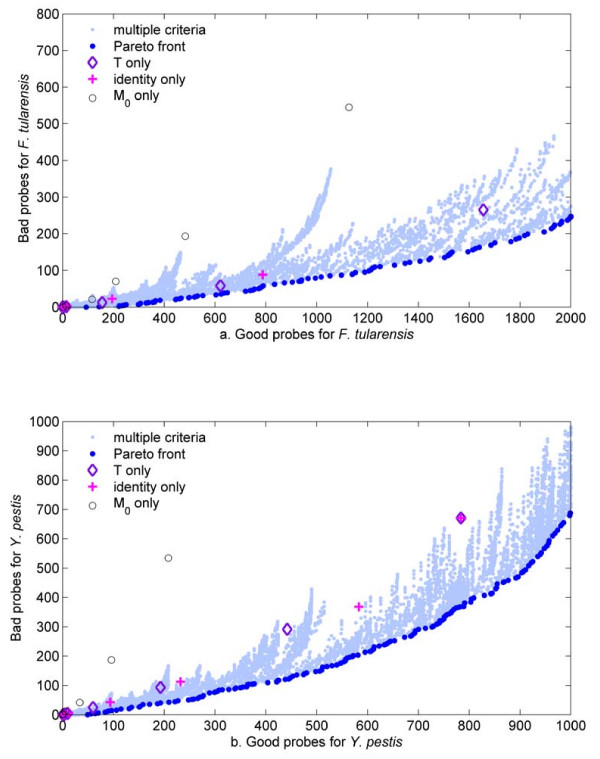
**Good versus bad probes for different combinations of individual and multiple specificity criteria**. Each light blue dot represents a combination of thresholds for the multiple criteria (identity, *M*_0_, *M*_1_, *M*_2_, and *M*_3_). The Pareto optimal front, defined as the scattered points with the smallest number of bad probes for a specified number of good probes, is shown in dark blue. The scattered points corresponding to strict thresholds for each individual criterion are close to the Pareto optimal front, but quickly move away from it as the thresholds are relaxed.

Figure 5 shows the numbers of good and bad probes obtained by using different thresholds for each individual specificity criterion and different combinations of values for the multiple specificity criteria, for both *F. tularensis *and *Y. pestis*. One interesting observation is that the total number of good probes obtained for *Y. pestis *is much smaller than that for *F. tularensis*. This is not surprising. Given the larger availability of sequence information for organisms related to *Y. pestis*, we expect a larger proportion of *Y. pestis *probes to cross-hybridize with nontarget sequences, decreasing the number and proportion of good probes. The most significant observation is that, in both cases, there are very few scattered points for each individual criterion (*T*, *M*_0_, identity) and there are significant discrete changes in the numbers of probes for each of these few entries. This suggests that the use of individual specificity criteria provides limited flexibility to control for the potential number of probes. Conversely, the use of multiple specificity criteria provides an almost continuous spectrum of options. Since, in the probe selection process, we wish to maximize the number of good probes and simultaneously minimize the number of bad probes, we use the Pareto optimality principle [32] to select combinations of specificity parameters that optimize this dual objective. The Pareto optimal front contains solutions to a multiobjective optimization problem that are best in satisfying all of the objectives simultaneously. That means that there can be other solutions that are better in satisfying one or several objectives, but they must be worse than the Pareto optimal solution in satisfying the remaining objectives. In our case, the Pareto optimal front identifies the scattered points with the smallest number of bad probes for a given number of good probes. The scattered points for the individual criteria consistently drift away from the Pareto optimal front as the number of good probes increases. This trend further suggests that multiple specificity criteria should be used if one is interested in obtaining a larger number of good *in silico *probes, while simultaneously admitting fewer bad probes.

Table 4 shows the numbers of good, bad and total (including gray) probes that pass different combinations of specificity criteria. The results clearly indicate the observation made above that the use of individual specificity criteria (*T*, identity, *M*_0_, etc.) does not provide fine control over the number of *in silico *fingerprints. Some selections provide either too few or too many probes. For example, a selection of *T *= 10 yields 310 probes for *F. tularensis*, while a selection of *T *= 8 yields 4810 probes. Similar behavior is also observed for other individual criteria. For instance, while *M*_0 _*= *16 yields 1580 probes, *M*_0 _= 17 yields 4254. Such large changes in the number of probes with small changes in specificity thresholds are not desirable in experimental testing. The customized microarray platform (Agilent Technologies [33]) that will be used for experimental testing consists of a slide with eight arrays. Though each array contains 15000 features, the inclusion of controls and at least five replicates per probe limits the actual number of probes on each array to around 2000. Using multiple criteria (first three rows in Table 4), however, we can achieve the desired number of probes by adjusting the thresholds, as small changes in individual thresholds do not lead to large differences in the number of *in silico *fingerprints.

**Table 4:** The numbers of good, bad and total probes (including gray) obtained for different specificity thresholds

**Thresholds for specificity criteria**	***F. tularensis***			***Y. pestis***		
	
	**Good**	**Bad**	**Total**	**Good**	**Bad**	**Total**
**Identity = 80%, *M*_0_-*M*_3 _= 18–22–26–30**	748	46	2028	215	44	836
**Identity = 80%, *M*_0_-*M*_3 _= 19–22–25–28**	820	63	2451	260	67	1042
**Identity = 82.5%, *M*_0_-*M*_3 _= 19–22–25–28**	1142	113	3532	359	111	1587
**Identity = 75%**	195	22	473	94	42	305
**Identity = 77.5%**	788	88	2019	232	112	833
**Identity = 80%**	2143	379	7199	583	368	2608
***T *= 8**	1656	265	4810	442	291	1895
***T *= 9**	621	58	1496	193	93	614
***T *= 10**	156	12	310	60	24	153
***M*_0 _= 16**	483	193	1580	96	187	628
***M*_0 _= 17**	1127	545	4254	208	534	1521
***M*_0 _= 18**	2632	1230	10665	491	1581	4040
***M*_1 _= 20**	418	119	1654	118	87	610
***M*_1 _= 21**	1857	523	7174	431	506	2581
***M*_1 _= 22**	3579	1226	14973	3758	1106	4952
***M*_2 _= 23**	486	90	1722	157	91	802
***M*_2 _= 24**	1646	383	6628	432	366	2567
***M*_2 _= 25**	3293	962	13967	771	751	4714
***M*_3 _= 25**	86	11	393	36	17	219
***M*_3 _= 26**	691	151	2866	223	160	1314
***M*_3 _= 27**	2026	533	8504	534	480	3305

## Discussion

### Effect of database size on specificity thresholds

The size of the target genome and the size of the database of nontarget sequences affect the selection of thresholds for contiguous and near-contiguous matches. For the size of target genomes and nontarget databases used in this paper, *M*_0 _= 18 appears to be an appropriate threshold, resulting in sufficient number of fingerprints for all the genomes in Table 3. However, as more and more nontarget sequences become available, it might not be possible to find unique 18 mers. Reed *et al*. [28] present a model to estimate the quality of a probe based on the size of the database of nontarget sequences. According to this model, the probability of finding a unique 18 mer is close to zero when the size of database is 100 Gb. As the size of sequence databases is quickly approaching this number, it might be necessary to use higher thresholds for *M*_0 _and other specificity parameters.

### Limitations of using BLAST

Even though BLAST is among the best methods for sequence comparisons against large databases like the *nt *database, BLAST has its limitations. BLAST is a heuristic approach that first finds short, exact, "anchor" matches and extends the alignments around these exact matches. The size of these exact matches, termed word size, is given by the input parameter *W*. In the *blastn *program, used for BLAST searches against large sequence databases, the default value for *W *is 11 and the smallest possible value *W *can take is 7. Because every match reported by BLAST must include an exact match of length *W*, it imposes a lower bound on the values of the specificity thresholds *M*_0_, *M*_1_, *M*_2_, and *M*_3_. For any given *W*, the smallest near-contiguous match with *i *mismatches that can be guaranteed to be reported by BLAST is given by *W*(*i*+1), therefore, the minimum allowable value for any *M*_*i *_is *W*(*i*+1)-1. Accordingly, the lowest thresholds of *M*_1_, *M*_2_, and *M*_3 _for *W *= 7 are 13, 20, and 27, respectively. Using lower thresholds for *M*_*i *_could result in some near contiguous matches of length *M*_*i *_not being reported by BLAST.

### Errors in sequence databases

TOFI currently uses the description provided in the header line of a FASTA sequence to determine if the sequence is a target or a nontarget sequence. Typographical errors or missing information in the NCBI sequence data can sometimes cause TOFI to treat a sequence from a target organism as a nontarget sequence, which can potentially lead to removal of good fingerprints.

The fingerprints designed by TOFI can only be as accurate as the sequence databases used for comparisons with nontarget sequences. However, identifying the errors and removing low-quality data from sequence databases is beyond the scope of the TOFI framework. The only complete solution for handling errors in sequence data is to use manually curated sequence databases that contain only high-quality sequences with accurate sequence descriptions.

### Extension to multiple genomes

The performance improvements in TOFI-beta pave the way for extending the pipeline to design probes common to a group of target genomes, e.g., multiple strains of a species and multiple species of a genus. The identification of common fingerprints is a more computationally intensive problem, as it requires the simultaneous analyses of multiple target genomes. In the future, we will extend the software pipeline to design sets of fingerprints common to multiple targets and sets unique to each target, taking maximum advantage of the shared sequences among the multiple genomes in order to reduce the overall computation time.

## Conclusion

The enhanced pipeline incorporates major algorithmic improvements, resulting in performance that is nearly up to five-times faster than the previous version of the pipeline. The use of multiple specificity criteria provides finer control over the number of resulting fingerprints. This is helpful in obtaining a larger number of *in silico *fingerprints than those obtained using individual criteria, and may be essential for the following three reasons: (1) as more and more genomic sequences become available, there will be significant *fingerprint erosion; *matches with the newly available sequences will eliminate some fingerprints, (2) certain near-neighbor organisms may have very similar sequences, so, in these cases, obtaining a larger number of potential *in silico *fingerprints for experimental testing would be desirable, and (3) due to the noisy nature of microarray experiments, redundancy is essential for confidence in the results. Therefore, it is desirable to start with a sufficiently large number of *in silico *fingerprints, and identify specific fingerprints based on experimental results with the target and a panel of nontarget sequences.

## Availability and requirements

• **Project name: **TOFI

• **Project home page: **

• **Operating systems: **Linux

• **Programming Language: **Perl

• **Other Requirements: **mpiBLAST 1.4.0 or higher, MUMmer 3.19 or higher, and OMP developer edition

## Authors' contributions

RVS conceived and implemented the algorithmic improvements in TOFI-beta. KK developed the user interface for TOFI-beta and modules for running OMP. NZ analyzed the simulation data for specificity threshold selection. JR conceived the project and provided overall project guidance. RVS, NZ and JR were the main writers of the manuscript.

## References

[B1] Slezak T, Kuczmarski T, Ott L, Torres C, Medeiros D, Smith J, Truitt B, Mulakken N, Lam M, Vitalis E, Zemla A, Zhou CE, Gardner S (2003). Comparative genomics tools applied to bioterrorism defence. Brief Bioinform.

[B2] Kaderali L, Schliep A (2002). Selecting signature oligonucleotides to identify organisms using DNA arrays. Bioinformatics.

[B3] Phillippy AM, Mason JA, Ayanbule K, Sommer DD, Taviani E, Huq A, Colwell RR, Knight IT, Salzberg SL (2007). Comprehensive DNA signature discovery and validation. PLoS Comput Biol.

[B4] Tembe W, Zavaljevski N, Bode E, Chase C, Geyer J, Wasieloski L, Benson G, Reifman J (2007). Oligonucleotide fingerprint identification for microarray-based pathogen diagnostic assays. Bioinformatics.

[B5] Loy A, Bodrossy L (2006). Highly parallel microbial diagnostics using oligonucleotide microarrays. Clin Chim Acta.

[B6] Wilson WJ, Strout CL, DeSantis TZ, Stilwell JL, Carrano AV, Andersen GL (2002). Sequence-specific identification of 18 pathogenic microorganisms using microarray technology. Mol Cell Probes.

[B7] Wang D, Urisman A, Liu YT, Springer M, Ksiazek TG, Erdman DD, Mardis ER, Hickenbotham M, Magrini V, Eldred J, Latreille JP, Wilson RK, Ganem D, DeRisi JL (2003). Viral discovery and sequence recovery using DNA microarrays. PLoS Biol.

[B8] Palacios G, Quan PL, Jabado OJ, Conlan S, Hirschberg DL, Liu Y, Zhai J, Renwick N, Hui J, Hegyi H, Grolla A, Strong JE, Towner JS, Geisbert TW, Jahrling PB, Buchen-Osmond C, Ellerbrok H, Sanchez-Seco MP, Lussier Y, Formenty P, Nichol MS, Feldmann H, Briese T, Lipkin WI (2007). Panmicrobial oligonucleotide array for diagnosis of infectious diseases. Emerg Infect Dis.

[B9] CGH Whole-Genome & Custom Fine-Tiling Microarrays and Services. http://www.nimblegen.com/products/cgh/index.html.

[B10] Nordberg EK (2005). YODA: selecting signature oligonucleotides. Bioinformatics.

[B11] Rimour S, Hill D, Militon C, Peyret P (2005). GoArrays: highly dynamic and efficient microarray probe design. Bioinformatics.

[B12] Chen H, Sharp BM (2002). Oliz, a suite of Perl scripts that assist in the design of microarrays using 50mer oligonucleotides from the 3' untranslated region. BMC Bioinformatics.

[B13] Wernersson R, Nielsen HB (2005). OligoWiz 2.0--integrating sequence feature annotation into the design of microarray probes. Nucleic Acids Res.

[B14] Rahmann S (2003). Fast and sensitive probe selection for DNA chips using jumps in matching statistics. Proc IEEE Comput Soc Bioinform Conf.

[B15] Rahmann S (2003). Fast large scale oligonucleotide selection using the longest common factor approach. J Bioinform Comput Biol.

[B16] Loy A, Maixner F, Wagner M, Horn M (2007). probeBase--an online resource for rRNA-targeted oligonucleotide probes: new features 2007. Nucleic Acids Res.

[B17] Volokhov D, Pomerantsev A, Kivovich V, Rasooly A, Chizhikov V (2004). Identification of Bacillus anthracis by multiprobe microarray hybridization. Diagn Microbiol Infect Dis.

[B18] Zhu LX, Zhang ZW, Wang C, Yang HW, Jiang D, Zhang Q, Mitchelson K, Cheng J (2007). Use of a DNA microarray for simultaneous detection of antibiotic resistance genes among staphylococcal clinical isolates. J Clin Microbiol.

[B19] Putonti C, Chumakov S, Mitra R, Fox GE, Willson RC, Fofanov Y (2006). Human-blind probes and primers for dengue virus identification. Febs J.

[B20] He Z, Wu L, Li X, Fields MW, Zhou J (2005). Empirical establishment of oligonucleotide probe design criteria. Appl Environ Microbiol.

[B21] Kurtz S, Phillippy A, Delcher AL, Smoot M, Shumway M, Antonescu C, Salzberg SL (2004). Versatile and open software for comparing large genomes. Genome Biol.

[B22] OMP Developer Edition. http://www.dnasoftware.com/Products/OMP_DE/index.htm.

[B23] SantaLucia J, Hicks D (2004). The thermodynamics of DNA structural motifs. Annu Rev Biophys Biomol Struct.

[B24] Altschul SF, Gish W, Miller W, Myers EW, Lipman DJ (1990). Basic local alignment search tool. J Mol Biol.

[B25] Darling A, Carey L, Feng W (2003). The Design, Implementation, and Evaluation of mpiBLAST. 4th International Conference on Linux Clusters: The HPC Revolution 2003 in conjunction with the ClusterWorld Conference & Expo.

[B26] Li X, He Z, Zhou J (2005). Selection of optimal oligonucleotide probes for microarrays using multiple criteria, global alignment and parameter estimation. Nucleic Acids Res.

[B27] Kane MD, Jatkoe TA, Stumpf CR, Lu J, Thomas JD, Madore SJ (2000). Assessment of the sensitivity and specificity of oligonucleotide (50mer) microarrays. Nucleic Acids Res.

[B28] Reed C, Fofanov V, Putonti C, Chumakov S, Slezak T, Fofanov Y (2007). Effect of the mutation rate and background size on the quality of pathogen identification. Bioinformatics.

[B29] Chou CC, Lee TT, Chen CH, Hsiao HY, Lin YL, Ho MS, Yang PC, Peck K (2006). Design of microarray probes for virus identification and detection of emerging viruses at the genus level. BMC Bioinformatics.

[B30] Liebich J, Schadt CW, Chong SC, He Z, Rhee SK, Zhou J (2006). Improvement of oligonucleotide probe design criteria for functional gene microarrays in environmental applications. Appl Environ Microbiol.

[B31] JGI Microbial Genomes. http://genome.jgi-psf.org/mic_cur1.html.

[B32] Censor Y (1977). Pareto Optimality in Multiobjective Problems. Appl Math Optimiz.

[B33] Agilent DNA microarrays. http://www.chem.agilent.com/Scripts/PCol.asp?lPage=494.

